# Beyond the ‘purple drank’: Study of promethazine abuse according to the European Medicines Agency adverse drug reaction reports

**DOI:** 10.1177/0269881120959615

**Published:** 2021-01-10

**Authors:** Stefania Chiappini, Fabrizio Schifano, John Martin Corkery, Amira Guirguis

**Affiliations:** 1 Psychopharmacology, Drug Misuse and Novel Psychoactive Substances Research Unit, University of Hertfordshire, Hatfield, UK; 2 Swansea University Medical School, Institute of Life Sciences 2, Swansea University, Swansea, UK

**Keywords:** Promethazine, promethazine misuse, drug abuse, purple drank, sizzurp, codeine/promethazine, adverse drug reactions, pharmacovigilance

## Abstract

**Background:**

Promethazine is a medicinal product, available on its own or in combination with other ingredients including dextromethorphan, paracetamol and/or expectorants. Anecdotal reports have however indicated that promethazine may have a misuse potential, especially in adolescents.

**Objective:**

We here aimed at studying how this phenomenon has been reported to the European Monitoring Agency Adverse Drug Reactions database.

**Methods:**

After a formal request to the European Monitoring Agency, the promethazine-specific dataset has been studied, performing a descriptive analysis of misuse/abuse/dependence-related adverse drug reaction reports. The study was approved by the University of Hertfordshire (LMS/PGR/UH/03234).

**Results:**

The analysis of promethazine data showed increasing levels of misuse/abuse/ dependence issues over time (2003–2019). Out of a total number of 1543 cases of adverse drug reactions, the abuse/misuse/dependence-related cases reported were 557, with ‘drug abuse’ (300/557: 53.8%) and ‘intentional product misuse’ (117/557: 21.0%). being the most represented adverse drug reactions. A high number of fatalities were described (310/557: 55.6%), mostly recorded as ‘drug toxicity/drug abuse’ cases, with opiates/opioids having been the most commonly reported concomitant drugs used.

**Conclusion:**

Anecdotal promethazine misuse/abuse reports have been confirmed by European Monitoring Agency data. Promethazine misuse/abuse appears to be an alarming issue, being associated with drug-related fatalities. Thus, healthcare professionals should be warned about a possible misuse of promethazine and be vigilant, as in some countries medicinal products containing promethazine can be purchased over the counter. Since promethazine is often available in association with opioids, its abuse may be considered a public health issue, with huge implications for clinical practice.

## Introduction

### Pharmacological properties of promethazine

Promethazine is a phenothiazine derivative and a histamine (H1) receptor antagonist that is commonly used for symptomatic relief of nausea and vomiting, for allergic conditions, motion sickness and common cold, and for short-term use treatment of insomnia in adults or as a paediatric sedative (EMC, 2019). It also acts as a direct antagonist at muscarinic (M1) and dopamine (D2) receptors (Cookson, 2018; Page et al., 2009; Sharma and Hamelin, 2003). Promethazine hydrochloride is well absorbed from the gastrointestinal tract, with an average of 88% of the dose absorbed after oral administration, and clinical effects appearing within 20 min after intake whilst lasting 4–6 h. The plasma half-life is approximately 7–14 h (Burns and Boyer, 2013). It is classified as a first-generation antihistamine molecule which, compared with second-generation antihistamines, easily penetrates the blood-brain barrier and is associated with adverse effects such as moderate/intense sedation (Jensen et al., 2017). Thus, promethazine might be used in rapid (acute) tranquilization due to its blocking action at H1 and M receptors (Cookson, 2018). Toxicity might result in severe impairment of cognitive and psychomotor functions due to central nervous system (CNS) depression/reduced levels of consciousness, and may cause fatalities (Jensen et al., 2017).

### Availability of promethazine

Promethazine is a medicinal product available on its own or in combination with other ingredients, including dextromethorphan, paracetamol and/or expectorants. With regards to promethazine availability, there are wide variations across countries. In fact, in some European countries such as France, Italy and the UK, but also outside Europe, some medicinal products containing promethazine can be purchased over-the-counter (OTC). Promethazine was restricted from an OTC status to prescription only in Denmark as of December 2014. This restriction was partly based on increasing enquiries regarding promethazine exposures to the Danish Poison and Information Centre (DPIC), highlighting its abuse potential and subsequent observations of side-effects after ingestion of standard doses. In Australia, promethazine is available as an OTC medication either alone as a tablet or as a liquid preparation or in combination with paracetamol and codeine phosphate as a syrup (Sharma and Hamelin, 2003). In the USA, some cough preparations containing no more than 200 mg of codeine per 100 mL are considered Schedule V controlled substances (Drug Enforcement Administration (DEA), 2020), purportedly possessing the lowest potential for both misuse and development of a Substance Use Disorder (SUD).

### Pharming

Recently, the potential for misuse of medications that are not already controlled has been reported worldwide (Reeves et al., 2015). ‘Pharming’ is a phenomenon involving the non-medical use of prescription (pain relievers, tranquilisers, stimulants, sedatives) and OTC drugs, including cough and cold preparations, particularly those containing dextromethorphan and promethazine (Burns and Boyer, 2013; Jouanjus et al., 2018; Levine, 2007; McCarthy, 2007; National Institute on Drug Abuse (NIDA), 2014). The abuse of these compounds may be facilitated by their easy accessibility, low cost, decreased perception of potential for harm and growing social acceptance (Burns and Boyer, 2013; Levine, 2007; McCarthy, 2007; Substance Abuse Mental Health Services Administration (SAMHSA), 2008). In the current drug abuse scenario, young people/youths have been described abusing with a range of prescription or OTC medicines (Soussan et al., 2018), with these molecules possibly being ingested at high/super-high dosages (Orsolini et al., 2015; Schifano, 2020) and at times in combination with alcohol/remaining recreational substances (Levine, 2007)

### Recreational use of promethazine

Centrally acting antihistamines have long been known to induce physical and psychological dependence (Clatts et al., 2010; Gracious et al., 2010; Page et al., 2009; Saatcioglu and Evren, 2005; Thomas et al., 2008). During the past 15 years the abuse of promethazine, especially from OTC and prescription cough and cold medicines and at higher-than-recommended dosages, has been reported (NIDA, 2014). Consistent with this, during the last decade the yearly number of promethazine-related overdose cases in Sweden has increased from 100 to nearly 700 and its sales have increased threefold (Höjer and Tellerup, 2018). Also, according to the 2018 Annual Report of the American Association of Poison Control Centers’ National Poison Data System (NPDS), antihistamines accounted for the seventh most frequently involved substance in human exposures, resulting in the third type of substance category for rate of exposure increase (Gummin et al., 2019). In Denmark, the number of registered antihistamine exposures increased during 2007–2013, with first-generation antihistamines, specifically promethazine, having been responsible for most exposures and related fatalities (Jensen et al., 2017). Also, in New Zealand a number of promethazine poisoning cases (57/199 patients) were identified from a database of poisoning admissions to a regional toxicology service (Bergman and Wallman, 1998).

#### Effects

Promethazine reported effects mostly include a range of CNS side effects, such as: confusion; disorientation; drowsiness; cardiovascular symptoms; and respiratory depression (Burns and Boyer, 2013; Höjer and Tellerup, 2018; Page et al., 2009; Tsay et al., 2015). A clear abuse potential has been observed and reported for first-generation antihistamines such as promethazine and cyclizine, due to: calming and sedating effects (Cookson, 2018; Jensen et al., 2017) and enhancement of other co-ingested substances, and especially those interacting with gamma-amino-butyric acid (GABA), opiate and muscarinic acetylcholine receptors (Speeg et al., 1981), leading to hallucinogenic experiences (Clatts et al., 2010; Jensen et al., 2017; Lynch et al., 2015). Indeed, an anti-cholinergic toxidrome with hyperthermia, flushing, tachycardia, dry mucosa, mydriasis, urinary retention and gastrointestinal dysmotility has been described (Burns and Boyer, 2013). In these cases, the mental state alteration is characterised by agitated delirium, with abnormal thoughts, irritability, distressing visual hallucinations, disorganised behaviour and insomnia (Agnich et al., 2013; Cowen, 1979; Dollberg et al., 1989; Höjer and Tellerup, 2018; Leak and Carroll, 1967; Page et al., 2009; Scott et al., 2007; Timnak et al., 2004). With the promethazine-codeine cough syrup, ‘euphoric feelings’, ‘relaxation’, ‘slight giddiness and disorienting’, and ‘nice hallucinations’ have been described as well (Bluelight.org [2020]; Erowid.org [2020]). Reported side-effects include drowsiness, fatigue, loss of coordination, constipation and urinary retention (Elwood, 2001).

#### Vulnerable categories

An increasing trend in abuse of promethazine in co-formulation with some components of OTC cough medications has been reported in young adult populations (Carney et al., 2018; Carr, 2006; Höjer and Tellerup, 2018) since the late 1990s, particularly in the southern USA (Carr, 2006; Elwood, 2001). Codeine and promethazine were recorded as the most requested psychoactive drugs by adolescents/young adults in community pharmacies (Delcourt et al., 2017).

Known by the street names ‘lean’, ‘drank’, ‘barre’, ‘purple stuff’, ‘syrup’ and ‘sizzurp’ (Agnich et al., 2013; NIDA, 2014; Peters et al., 2007), the abuse of promethazine mixed with opioids and other sedatives (e.g. alcohol) in a purple colour drink (‘purple drank’) (Agnich et al., 2013; Burns and Boyer, 2013; Cherian et al., 2018; NIDA, 2014) has become widely popular (Agnich et al., 2013) after a rap artist/producer created a genre of music, called ‘screw music', inspired by intoxication on codeine and promethazine (Peters et al., 2007). Social media data, e.g. public posts and related hashtags on Instagram, provided additional insights into codeine abuse, depicting its common misuse combined with promethazine and soda/carbonated drinks (Cherian et al., 2018). ‘Purple drank’ has been noted for its euphoric effects and its easy accessibility (Elwood, 2001; Peters et al., 2007). ‘Lean’ creates a distinct (Jakubowski et al., 2018) feeling of euphoria and extreme relaxation (Cookson, 2018), dream-like feelings and a vivid sensation of floating away from the physical body (Peters et al., 2007).

Although at times preferred to other substances, such as benzodiazepines, to treat anxiety and sleep disorders in substance-dependent patients, promethazine has been reported to be misused among people with either opioid dependence (Agnich et al., 2013; Dahlman et al., 2016; Elwood, 2001; Lynch et al., 2015; Shapiro et al., 2013) or a substance abuse condition (Shapiro et al., 2013; Tang et al., 2012). A Hong Kong study reported a total of 63 patients with a diagnosis of cough mixture abuse; 89% were adult males with a mean age of onset of the abuse itself of 20 (±5) years. These subjects had received a diagnosis of substance-induced psychotic disorder (67%), schizophrenia (19%), depressive disorder (11%) and dysthymia (10%), with most cases having presented with a polysubstance misuse (Tang et al., 2012). Due to the opioid-coformulation, the promethazine-codeine cough mixture might lead to addiction (NIDA, 2014). The mixture may present a high risk of occurrence of near misses due to its central effect of respiratory depression, which is greatly increased by association with alcohol or other CNS depressants, such as sedatives/hypnotics, narcotic analgesics, general anaesthetics and tricyclic antidepressants (Burns and Boyer, 2013; Elwood, 2001; NIDA, 2014; Shapiro et al., 2013; Tsay et al., 2015). The concomitant use of promethazine and other depressant drugs should not be underestimated, as they might together contribute to and/or cause fatalities (Corkery et al., 2020).

## Aim of the study

We were interested here in studying how promethazine abuse was reported to the European Medicines Agency (EMA) adverse drug reactions (ADRs) database. We aimed to analyse promethazine misuse/abuse/dependence/withdrawal data. Where possible, in order to have a better understanding, information/data related to diagnosis, concomitant drugs, administration and dosages were also examined.

## Methods

### Source of data

The EMA through EudraVigilance (EV) manages and analyses information on suspected ADRs to medicines authorised in the European Economic Area (EEA), according to Directive 2001/83/EC and Regulation (EC) No 726/2004 (EMA, 2017). Since November 2017, EV launched extensive Web access to data on suspected ADRs and the possibilities for academic research institutions to request a more extensive dataset for the purposes of health research (Postigo et al., 2018). Thus, access to data regarding cases of promethazine abuse, misuse, dependence and withdrawal was obtained by application to the voluntary reporting system of ADRs of the EMA. After 3 months all data comprised all of the ADRs reported during the years from 2003 up to June 2019, presented as large Excel files divided into information sections reporting in a standardised format according to the Medical Dictionary for Regulatory Activities (MedDRA), were sent through a hyperlink (MedDra, 2018). Such listings showed all information related to the ADR, the patient, the drug, the reporter and the diagnosis. An ADR was considered a voluntary and unsolicited communication reported by both Regulatory Authorities of the EU Member States where the reaction occurred and/or by the Marketing Authorisation Holders for those ADRs occurring outside the EEA (EMA, 2010). Only ‘suspect’ ADRs were selected, meaning promethazine was considered as ‘suspected’ for the reaction reported. Regarding the content of the ADR, according to the standardised MedDRA Query (SMQ) (MedDRA, 2018), the following ADRs: dependence, drug abuse, drug abuser, drug dependence, drug withdrawal convulsions, drug withdrawal syndrome, drug withdrawal neonatal syndrome, intentional product misuse, intentional product use issue, substance abuse, substance abuser, substance use and withdrawal syndrome were selected. Those ADRs were defined in accordance with MedDRA in line with previous studies on the EV dataset (Chiappini et al., 2020; MedDRA, 2018; Schifano and Chiappini, 2018a, 2018b; Schifano et al., 2019a, 2019b). Specifically, *abuse* was here defined as the intentional, non-therapeutic use by a patient or consumer of a product, OTC or prescription, for a perceived reward or desired non-therapeutic effect including, but not limited to, getting high (euphoria) (MedDRA, 2018). *Misuse* was meant as the intentional use for a therapeutic purpose by a patient or consumer of a product, OTC or prescription, other than as prescribed or not in accordance with the authorised product information (MedDRA, 2018). Finally, *non-medical use* referred here to the use of a prescription drug, whether obtained by prescription or otherwise, other than in the manner or for the time period prescribed, or by a person for whom the drug was not prescribed (United Nations Office on Drugs and Crime (UNODC), 2013).

### Analysis of data

We retrospectively analysed all reported ADRs which were available in the EV database. ADR reports were analysed with respect to age and gender of patient/consumer, source/reporter country (EEA or non-EEA) and reporter qualification (i.e. pharmacist, physician); type of ADR; seriousness (fatal, recovered, resolved outcomes); promethazine dosage; possible concomitant drug(s); and diagnosis/reporter’s comments, if recorded. The descriptive analysis included cases of overdoses, suicides, and fatalities. In the dataset, each case report may refer to one or more reporter; one or more ADR(s); as well as to one or more medicinal product(s). Therefore, a case may be represented by more than one row in the other line listings. The received data files were searched for duplicates by report ID through the ‘EV Local Report Number’, which unequivocally identified an individual case. Thus, the number of suspected ADRs appeared to be different from the number of case reports as one case report might refer to several suspected ADRs. Moreover, the number of patients was different from the number of case reports as a patient might have been described in more than one case. Finally, numbers of ADRs differed from those referring to case reports/single patients since different reporters/senders could have independently flagged the same ADR to the EMA. The descriptive analysis included here a study of the dataset (EMA, 2017) performed through IBM SPSS Statistics software (version 26).

### Ethical issues

In compliance with applicable Personal Data Protection legislation (Regulation (EC) No 45/2001 and Regulation (EC) No 1049/2001, the protection of privacy and integrity of individuals was guaranteed, and in order to safeguard the identity of individuals certain data elements, including names/identifiers or country-specific information were not disclosed by the EMA (EMA, 2013, 2017). The study was ethically approved in March 2018 by the University of Hertfordshire Ethics’ Committee, with reference number LMS/PGR/UH/03234.

## Results

**Figure 1.**
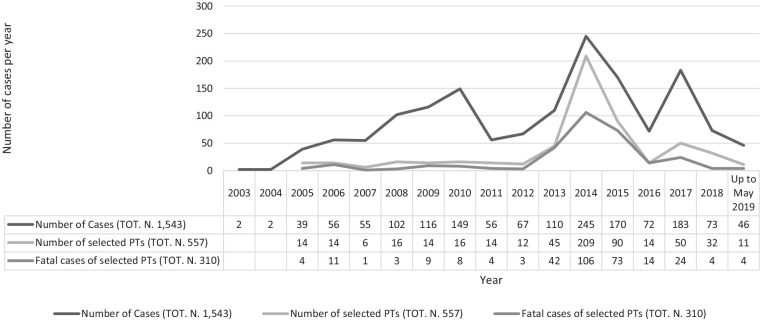
Number of promethazine abuse/misuse/dependence/withdrawal cases reported by year in the EudraVigilance (EV) dataset.

**Figure 2.**
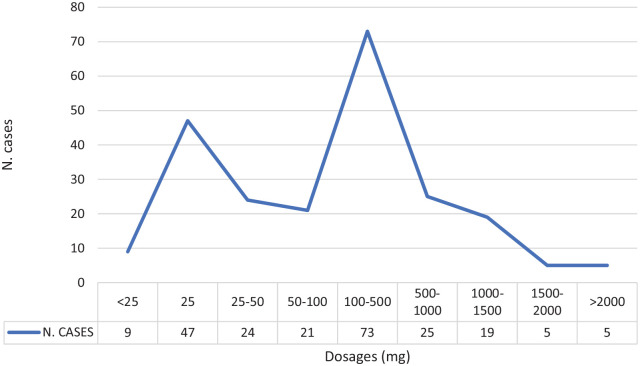
Most represented dosages reported among all promethazine abuse/misuse/dependence/withdrawal cases recorded by the EudraVigilance (EV) dataset.

The dataset obtained reported a total of 11,796 ADRs, correspond-ing to 1,543 single cases recorded during years 2003–2019 (Table 1). Safety reports were all voluntarily submitted, mostly from pharmaceutical companies (1,136/1,543: 73.6%) and regulatory authorities (398/1,543: 25.8%). Reports were irregularly reported over time, with spikes in 2010, 2014 and 2017 (Figure 1). Countries which reported most promethazine-related abuse/misuse/dependence cases included: the USA (805/1,543: 52.10%); Germany (299/1,543: 19.4%); Japan (162/1,543: 10.5%), Sweden (67/1,543: 4.3%), France (43/1,543: 2.8%) and Australia (42/1,543: 2.7%). Out of a total of 1543 cases, ‘suspect’ abuse/misuse/dependence-related cases were 557 (36.1%) and, among them, according to the preferred terms (PTs), most recorded reactions were abuse/misuse-related ADRs (458/557: 82.2%), specifically ‘drug abuse’ (300/557: 53.8%) and ‘intentional product misuse’ (117/557: 21.0%) (Table 1). ‘Suspect’ selected cases and related fatalities annually showed the same trend overall (Figure 1). Most typically, subjects reported were adult (19–64 years) and specifically of the youngest group (19–25 years); males were slightly more frequently recorded than females (M/F: 235/461: 0.51) (Table 1). Where all information was recorded, promethazine was reported as ingested alone in 74 cases, with supratherapeutic dosages, up to 2500 mg, in four cases. In combination with other drugs, most cases were associated with a 100–500 mg promethazine dose, with the maximum dosage recorded having been 8000 mg (Figure 2). The most common administration route was oral (*n* = 292), although the intramuscular (*n* = 8) and the parenteral (*n* = 19) ones were reported as well, see Table 1).

**Table 1.** Analysis of promethazine abuse/misuse/dependence/withdrawal cases recorded by EudraVigilance (EV) during years 2003–2019.

	Individual cases (% of total within parentheses)
Total abuse/misuse/dependence cases	1543 Single cases; number of ADRs :11,796
Age range	Adult (19–64 years): 648 (648/1,543: 42.0%) - mean age: 31.8 years (SD 26.55–37.05)
	Adolescent (10–18 years): 23 (23/1,543: 1.5%) – mean age: 15.9 years (SD 14.3–17.77)
	Elderly (>65 years): 25 (25/1,543: 1.6%) – mean age: 72.3 years (SD 70.85–73.7)
	Neonatal (hours–days) 14 (14/1,543: 0.9%) – mean age: 24 h (SD 16.6–27.4)
	Infant (months–1 year): 7 (7/1,543: 0.45%) – mean age: 10 months (SD 7–13)
	Child (<10 years): 4 (4: 1,543: 0.35%) – mean age: 5 years (SD 3.6–6.3)
	Unknown: 822 (822/1,543: 53.2%)
Male/female	235/461: 0.51
Most represented abuse/misuse/dependence-related ADRs according to the PTs:	557 (557/1,543: 36.1%)
Abuse-related ADRs	458 (458/557: 82.2%)
Drug abuse	300
Drug abuser	15
Drug diversion	1
Intentional product misuse	117
Intentional product use issue	9
Substance abuse	11
Substance abuser	3
Substance use	2
Dependence-related ADRs	44 (44/557: 7.9%)
Dependence	4
Drug dependence	39
Substance dependence	1
Withdrawal-related ADRs	55 (55/557: 9.8%)
Withdrawal syndrome	19
Drug withdrawal convulsions	1
Drug withdrawal neonatal syndrome	18
Drug withdrawal syndrome	17
Outcome	Fatal 310 (310/557: 55.6%)
	Unknown 161 (161/557: 28.9%)
	Recovered/resolved 55 (55/557: 9.9%)
	Recovering/resolving 18 (18/557: 3.3%)
	Not recovered/not resolved 13 (13/557: 2.3%)
Promethazine-cases alone	74 (with maximum dosage 2500 mg)
Promethazine-cases with other drugs	Most cases (122) were over 100 mg (with maximum dosage 8000 mg)
Most common psychoactive substances used	Alcohol: 114
	Cocaine: 68
	Cannabis: 16
	Ketamine: 4
	Amphetamine: 1
Most common prescription drugs used	Opioids: 1187
	Benzodiazepines: 914
	Antidepressants: 871
	Antipsychotics: 437
	Z-drugs: 222
	Mood stabilisers: 197

ADR: adverse drug reaction; PTs: preferred terms; SD: standard deviation.

Concomitantly used drugs recorded were opioids (e.g. oxycodone and fentanyl), benzodiazepines (e.g. diazepam, alprazolam and lorazepam) and antidepressants (e.g. citalopram, venlafaxine and amitriptyline), whilst recreational drugs most represented were alcohol and cocaine (Table 1). Most reported diagnoses, according to the *Diagnostic and Statistical Manual of Mental Disorders 5th edition* (DSM-V; American Psychiatric Association (APA), 2013) related to: mood disorders, e.g. depression/depressed mood/major depression (68 cases) and bipolar disorder (14 cases); anxiety/anxiety disorders (23 cases); alcohol abuse/alcoholic/alcoholism (26 cases); and schizophrenia (14 cases). The outcome of the reactions reported included: ‘fatal’ (*n*=310/557; 55.6%) and secondarily ‘recovered/resolved’ (*n*=73; 15.2%) (Table 1). Some 27 cases were related to suicidal or self-harm behaviour, being recorded as ‘suicidal attempt/suicidal ideation’ (*n*=24), ‘self-injurious ideation’ (*n*=1), and ‘suicide attempt’ (*n*=2). Fatalities mostly involved adult females; in these cases, most typical ADRs recorded were ‘drug abuse’ (*n*=228) and ‘intentional product misuse’ (*n*=77) (Table 2). Opiates/opioids were the most recorded concomitant drugs (*n*=356), with methadone being the most represented (*n*=103), followed by antidepressants (*n*=221) and benzodiazepines (*n*=141). Among illicit drugs, heroin appeared to be the most represented (Table 2).

**Table 2.** Analysis of fatal promethazine abuse/misuse/dependence/withdrawal cases recorded by EudraVigilance (EV), 2003–2019.

Fatal cases on abuse/misuse/dependence/withdrawal reactions	310 (310/557 = 55.6%)
Age-range	
Adult	303 (97.7%)
Adolescent	7 (2.3%)
Elderly (>65 years)	–
Neonatal	–
Infant	–
Child	–
Gender	M 103 (33.2%)
	F 177 (57.1%)
	Unknown 30 (9.7%)
Most recorded PTs	
Drug abuse/drug abuser/substance abuse	228/3/6
Intentional product misuse/Intentional product use issue	77/3
Drug dependence	1
Reported death code	
Intentional overdose	1
Overdose	4
Completed suicide/suicide	7
Drug toxicity/drug abuse	197
Toxicity to various agents	48
Intentional product misuse	41
Respiratory depression	5
Pneumonia	1
Cardiac arrest	10
Drug dependence	1
Most reported concomitant drugs	
**Opioids**	**356**
Methadone	103
Dihydrocodeine	1
Codeine	32
Fentanyl	44
Oxycodone	63
Morphine	55
Hydrocodone	33
Hydromorphone	3
Tramadol	22
**Antidepressants**	**221**
Duloxetine	1
Escitalopram	1
Sertraline	16
Paroxetine	34
Trazodone	15
Mirtazapine	33
Fluoxetine	24
Venlafaxine	9
Bupropion	1
Amitriptyline	35
Nortriptyline	6
Clomipramine	5
Citalopram	41
**Benzodiazepines**	**141**
Lorazepam	3
Temazepam	6
Clonazepam	21
Diazepam	60
Flunitrazepam	1
Brotizolam	1
Alprazolam	42
Midazolam	4
Oxazepam	3
**Mood stabilisers**	**11**
Gabapentin	3
Topiramate	8
**Antipsychotics**	**24**
Olanzapine	1
Quetiapine	20
Haloperidol	1
Amisulpride	1
Levomepromazine	1
**Z-drugs**	**39**
Zolpidem	37
Zopiclone	2
**Illicit drugs**	
Amphetamine	15
Cocaine	40
Heroin	52
**Alcohol**	26

PTs: preferred terms.

## Discussion

Non-medical prescription drug use is a globally recognised problem, with severe adverse consequences. The current study provides a large amount of descriptive data highlighting that promethazine can be taken at high dosages, through non-approved administration routes, and in a setting of polydrug use (Hughes et al., 2016; Novak et al., 2016).

Promethazine misuse has been increasingly recorded since the early 2000s, consistently with data from US poison centres reporting antihistamine exposures rising in the period 2007–2013 (Jensen et al., 2017). This is not surprising, since the pharmacodynamic properties of promethazine underpins its sought-after central effects. In 2014, e.g. the highest peak here, the increase in promethazine misuse reporting was solely driven by the USA (245/245 cases; 100%). This may have been associated with intense media attention to the subject in that year (TMZ, 2014). The increments reported in 2010, 2014 and 2017 were then followed by decrements, which may have been related to not only reporting variabilities, but also to differences in sales of antihistamines following local/national drug abuse prevention campaigns (Klein-Schwartz, 2017). Overall, the USA appeared here to be the country most involved in reporting of promethazine ADRs. Several reasons may help in explaining this, including healthcare professionals’ awareness of prescription/OTC drug abuse (Agnich et al., 2013; Burns and Boyer, 2013; Lynch et al., 2015; Manchikanti et al., 2006; NIDA, 2014; Tsay et al., 2015) which, in turn, may have facilitated pharmacovigilance reporting. According to an 11-year period of the US National Poison Centers’ database, the annual rate of promethazine abuse/misuse per 100,000 US population doubled over time (Tsay et al., 2015). In the USA, promethazine abuse/misuse has been described in teenagers and young adults (Peters et al., 2003), methadone maintenance patients, heroin users and opioid-prescribed chronic pain patients (Lynch et al., 2015; Shapiro et al., 2013).

Consistent with previous observations (Agnich et al., 2013; Burns and Boyer, 2013; Carr, 2006; Elwood, 2001; Höjer and Tellerup, 2018; NIDA, 2014; Page et al., 2009; Parker et al., 2013; Peters et al., 2007), most recorded ADRs related here to either ‘drug abuse’ and ‘intentional product misuse’, involving young adult (19–25 years) males. Although considered a vulnerable category with respect to prescription drug abuse (Agnich et al., 2013; Burns and Boyer, 2013; Cherian et al., 2018; NIDA, 2014; Peters et al., 2007), adolescents (10–18 years) were here reported in only a minority (23/1543:1.5%) of cases.

Several drugs were here associated with promethazine, although opiates/opioids were the most represented molecules in related fatalities. This combination might intensify effects such as sedation and analgesia (Burns and Boyer, 2013; Lynch et al., 2015; Shapiro et al., 2013), and in the 1950s the association was medically used to reduce the dosage of opiates/opioids (McGee and Weiss, 1956). However, due to a range of adverse effects (Lynch et al., 2015) including lack of data supporting clinical efficacy (Richter and Burk, 1992) and an enhanced addiction potential of opioids (Lynch et al., 2015; Peters et al., 2007; Shapiro et al., 2013), the combination of promethazine/opioids has declined in popularity.

So far, the recreational intake of promethazine with opioids has been typically reported in cough mixtures containing codeine (Casati et al., 2012; Clatts et al., 2010; Elwood, 2001; Foley et al., 2015; Peters et al., 2007). However, current EMA data suggest that, apart from methadone, oxycodone and fentanyl were the most typical opiates/opiates identified in association with promethazine, possibly due to their recent popularity, especially in the USA (Manchikanti et al., 2018; Rose, 2018). In the promethazine fatalities (e.g. 310 cases) dataset analysed, codeine, fentanyl and oxycodone were respectively reported in 32, 44 and 63 cases. Consistent with current data, a concomitant use of opioids and promethazine has been described in various subpopulations, such as methadone maintenance patients, injecting drug users and chronic pain patients (Banta-Green et al., 2005; Lynch et al., 2015; Shapiro et al., 2013; Shields et al., 2007; Van Hout and Norman, 2016). Overall, there is now a high level of awareness of the abuse potential of antihistamine/opioid-containing products (Hou et al., 2011; Hughes et al., 1999; Wazaify et al., 2005). Hence, some countries are limiting both the medication pack size levels and the quantities of drugs sold in a single transaction, whilst also promoting non-injectable and non-inhaling formulations and changing some formulations’ OTC status (Cooper, 2013; Foley et al., 2015; Van Hout and Norman, 2016).

Benzodiazepines (e.g. diazepam, alprazolam and lorazepam) were here also frequently recorded, suggesting sedative synergistic effects in combination with promethazine (Lynch et al., 2015), and hence an increased risk of untoward clinical issues (McLellan and Turner, 2010). Other prescription drug categories recorded included antidepressants (most typically: citalopram and amitriptyline, respectively in 149/557 and 95/557 cases), which is consistent with the most typically recorded diagnoses, e.g. depressive (68 cases); bipolar (14 cases); and anxiety (23 cases) disorders. It is also possible that antidepressants were here ingested in combination with promethazine for sleep induction purposes. It is a reason for concern that promethazine ADRs, either alone or in combination, resulted in either fatal (50.6%) or ‘recovered/resolved’ outcomes (22.2%). This is consistent with previous promethazine misuse/abuse data, reporting both adverse clinical outcomes and high frequency of healthcare facility treatment (Bergman and Wallman, 1998; Ichikura et al., 2016; McLellan and Turner, 2010; Tsay et al., 2015). A list of possibly misused substances which might reinforce promethazine psychoactive effects and possibly increase its toxicity is included in Table 3.

**Table 3.** List of substances recorded in the literature as used in association with promethazine in order to increase its effects; prescription drugs are recorded according to the Neuroscience-based Nomenclature description (Nutt and Blier, 2016).

Psychoactive substances			
Description	Mode of action	Examples	Effects
Alcohol (Burns and Boyer, 2013; Jensen et al., 2017)	CNS depressant effects	‘Lean’, ‘sizzurp’, ‘purple drank’ and other street concoctions containing promethazine, codeine and alcohol, along with other potential sedatives	Euphoria, relaxation, ‘slight giddiness and disorienting’ and ‘nice hallucination’.
			It may increase, prolong or intensify promethazine sedative effect. It should be avoided in patients receiving promethazine.
Heroin and other illicit opioids (Dahlman et al., 2016; Tsay et al., 2015)	Opioid depressant effects		It may increase, prolong, or intensify promethazine sedative effect.
Prescription drugs			
Categories	Mode of action	Examples	Effects
Drugs for insomnia (Burns and Boyer, 2013)	Positive allosteric modulator (GABA-A receptor, benzodiazepine site)	Z-drugs: zaleplon, zolpidem, zopiclone, eszopiclone	They may increase, prolong or intensify promethazine sedative effect.
			They should be avoided or administered in reduced dosage to patients receiving promethazine.
Drugs for anxiety (Burns and Boyer, 2013; Lynch et al., 2015)	Positive allosteric modulator (GABA-A receptor, benzodiazepine site)	Benzodiazepines: alprazolam, chlordiazepoxide, clonazepam, clorazepate, diazepam, flunitrazepam, lorazepam, oxazepam	They may increase, prolong or intensify promethazine sedative action.
			They should be avoided or administered in reduced dosage to patients receiving promethazine.
Dextrometorphan (Tsay et al., 2015)	At high doses, acting as NMDA-receptor antagonist; dextrometorphan and its potent metabolite dextrorphan inhibit the excitatory amino acid and neurotransmitter glutamate in the brain, determining hallucinogenic and dissociative activities, which are recreationally searched	It might be combined with promethazine in cough-suppressant formulation	Dextrometorphan neurobehavioural effects are dose-related, starting from a mild to moderate stimulation with restlessness and euphoria (100–200 mg), to a dissociated state characterised by hallucinations, paranoia, perceptual distortions, delusional beliefs, ataxia and out-of-body experiences (‘robo-ing’/‘robo-copping’/‘robo-tripping’) (>1000 mg). In overdosage they might increase promethazine effects.
Drugs for depression (Burns and Boyer, 2013; Jensen et al., 2017): TCA	Multimodal action: reuptake inhibitor (SERT and NET), receptor antagonist (5-HT2)	Amitriptyline	Promethazine may increase, prolong or intensify their sedative effect. It should be avoided or administered in reduced dosage to patients receiving promethazine.
Anticholinergic drugs	Antagonist at cholinergic receptors	Hyoscine butyl bromide/scopolamine (Burns and Boyer, 2013)	Together with promethazine an anti-cholinergic toxidrome with hyperthermia, flushing, tachycardia, dry mucosa, mydriasis, urinary retention and gastrointestinal dysmotility may be seen. A typical mental status alteration with a dose-dependent agitated delirium characterised by abnormal thoughts, irritability, distressing visual hallucinations, disorganised behaviour and insomnia, has been described. These molecules should be avoided or administered in reduced dosage to patients receiving promethazine.
**Prescription opioids**			
	Codeine (Burns and Boyer, 2013; Jouanjus et al., 2018; Lynch et al., 2015; O Reilly et al., 2015; Shapiro et al., 2013)	‘Lean’, ‘sizzurp’, ‘purple drank’ and other street concoctions containing promethazine, codeine and alcohol, along with other potential sedatives	Euphoria, elation, analgesia and ‘liking’, with increased potential of addiction. Overdosage of codeine might determine respiratory depression (a decrease in respiratory rate and/or tidal volume, Cheyne–Stokes respiration and cyanosis), extreme somnolence progressing to stupor or coma, skeletal muscle flaccidity, cold and clammy skin, and sometimes bradycardia and hypotension. Opiate overdosage, particularly by the intravenous route, may be associated with apnoea, circulatory collapse, cardiac arrest and death. It should be avoided or administered in reduced dosage to patients receiving promethazine.
Other opioid analgesics (Burns and Boyer, 2013; Dahlman et al., 2016; Lynch et al., 2015; Shapiro et al., 2013)	Methadone		They may increase, prolong or intensify promethazine sedative effects. Opioids should be avoided or administered in reduced dosage to patients receiving promethazine.
			The addiction potential of opioids might be enhanced. Also, increasing life-threatening events, such as respiratory depression, overdose and prolongation of the QT interval, might be responsible for drug-related fatalities.

5-HT2: serotonin-2 receptor; CNS: central nervous system; D2: dopamine 2 receptor; GABA: gamma-amino-butyric acid; H1: histamine 1 receptor; NE: norepinephrine; NET: norepinephrine transporter; NMDA: N-methyl-D-aspartate; SERT: serotonin transporter; SSRI: selective-serotonin reuptake inhibitor; TCA: tricyclic antidepressant.

Finally, among abuse-related cases, neonates, infants and children were here reported as well. This is consistent with cases of unexpected infant deaths associated with use of cold medications, poor socioeconomic conditions (Rimsza and Newberry, 2008) and/or accidental ingestion (McLellan and Turner, 2010).

## Limitations

One of the limitations was given here by the descriptive nature of the study. Indeed, a comparator molecule, for a disproportionality analysis (Chiappini and Schifano, 2016; Schifano and Chiappini, 2018b) to be carried out, was not available due to data access limitations (EMA, 2017; Postigo et al., 2018). Furthermore, current data did not appear to show an increasing trend of abuse or misuse across the years. Since proper denominator figures (e.g. data on worldwide promethazine prescriptions) are not available, it was unclear if what observed was a substantial number of cases, indicative of clinically meaningful abuse potential, or a passing trend for promethazine misuse by a very small number of individuals. However, consistent with previous suggestions (Montastruc et al, 2011), the value of the current study was that its rationale was driven by a proper, detailed, pharmacodynamic hypothesis established on the basic properties of promethazine. Similar to remaining pharmacovigilance datasets (EMA, 2010; Evoy et al., 2019; Schifano and Chiappini, 2018a; Schifano et al., 2019a, 2019b; Schwan et al., 2010), focussing on the analysis of voluntary adverse events, a further limitation was given here by reliance on self-reporting and the likelihood of missing data occurrence. Moreover, although healthcare professionals have a main role in detection, assessment and spontaneous reporting of ADRs (Belton, 1997; Khalili et al., 2012), specific abuse/misuse/dependence issues relating to promethazine may have been underestimated, with only the most serious cases having been reported, hence the high rates of promethazine fatalities here recorded. Furthermore, although patients and their carers are allowed indeed to flag up an ADR, it is unlikely that they have spontaneously reported misusing events. Other factors, such as increased knowledge of promethazine misuse/abuse, may have resulted here in outcome reporting bias. There was also a potential for duplicate reports, with the same report submitted by the consumer, the healthcare professional and by the manufacturer as well, causing skewed study results. This may occur when a healthcare professional reported the same suspected ADR to both the national Regulatory Authority and the Marketing Authorisation Holder and they both reported subsequently to EV. However, the EV local report number which was here considered unequivocally identified an individual case. In addition, whilst this study represents one of the largest sample of promethazine abuse events published to date, the overall number of events is still relatively small, potentially limiting external validity. Case reports of suspected ADRs alone are not always sufficient to prove that a certain suspected reaction has indeed been caused by a specific medicine. This could be a symptom of another illness, or it could be associated with another medicinal product taken by the patient at the same time. The suspected ADRs were here presented using the PTs of the MedDRA Dictionary (Postigo et al., 2018). Thus, any case report should be considered together with all available data including case reports worldwide, clinical trials, epidemiological studies and toxicological investigations, in order to allow for robust conclusions. Finally, case reports reflect the information as provided to EV by the reporter, and not all data fields were completed for all reports.

## Conclusion

To the best of our understanding, the current study described in detail the largest sample ever of abuse, misuse and dependence issues related to promethazine, whilst focussing on a large multinational dataset, such as the EV. The substantial number of promethazine-related events identified over the years represents a pharmacovigilance signal which needs to be better investigated (Montastruc et al., 2011). Although the observed trend of promethazine abuse and misuse, especially in young adults, is not a new phenomenon, further details of the issue have been here provided and future studies will optimally identify the related risk factors, with these measures enabling policymakers and regulators to take action to detect and prevent such misusing practices (Jouanjus et al., 2019). A multicomponent approach is recommended, including monitoring drug utilisation, tracking users’ posts on social media, and exploring healthcare databases; this will enable performing proactive and effective post-marketing surveillance and pharmacovigilance approaches. This has proved to be a relevant, efficient and accurate strategy, for example with gabapentinoids, which have recently been rescheduled in the UK (Borg et al., 2011; Eickhoff et al., 2012; Jouanjus et al., 2019; Novak et al., 2016; Parker et al., 2013; Schifano and Chiappini, 2019; Throckmorton et al., 2018). In this context, the role of the Web is rapidly spreading, playing a significant role in the marketing, sale and distribution of drugs, hence facilitating continuous changes in drug scenarios (Orsolini et al., 2017). Indeed, over the last 10 years access to online pharmacies to purchase medicinal compounds has increased (Desai, 2016; Monteith and Glenn, 2018). On the other hand, professionals access the Web to gather data on emerging trends of drug abuse (Deluca et al., 2002; Orsolini et al., 2015; Schifano, 2020; Schifano et al., 2003).

OTC and prescription drug misuse is perceived to be a significantly under-recognised issue affecting a range of vulnerable individuals (Coombes and Cooper, 2019). However, controlling the problem of OTC misuse and abuse might be challenging, due to the need for achieving high levels of consumer safety whilst not restricting access to OTC products for those who continue to use them safely.

Staff training should be evaluated, in order for pharmacists to self-monitor care and use of medicines, to educate patients and intervene/support those experiencing problematic drug use (Elwood, 2001; GPC, 2019; Tobin et al., 2013; Wells et al., 2018). Record-keeping (Hou et al., 2011) and real time monitoring (Cairns et al., 2016) could be a method of restricting access to some OTC drugs and prevent ‘shopping’ from one pharmacy to another, and where these measures would result to be ineffective, regulatory interventions, e.g. drug re-scheduling, might be useful (Marsden et al., 2019; McDonough, 2016; Peacock et al., 2019). Also, prevention and early education on substance abuse in young teenagers are critical (Levine, 2007; Marsden et al., 2019). Finally, appropriate and specific clinical guidelines for the treatment of misuse, abuse and dependence on prescription or OTC drugs should be implemented (Fingleton et al., 2019).

## References

[bibr1-0269881120959615] Agnich LE Stogner JM Miller BL , et al. (2013) Purple drank prevalence and characteristics of misusers of codeine cough syrup mixtures. Addict Behav 38: 2445–2449. 23688907 10.1016/j.addbeh.2013.03.020

[bibr2-0269881120959615] American Psychiatric Association (2013) Diagnostic and Statistical Manual of Mental Disorders, 5th ed. Washington, DC: APA.

[bibr3-0269881120959615] Banta-Green CJ Jackson TR Hanrahan M , et al. (2005) Epidemiologic Trends in Drug Abuse: Proceedings of the Community Epidemiology Work Group. NIH Publication No. 05–5282. Bethesda, MD: National Institute on Drug Abuse, Division of Epidemiology, Services and Prevention Research; Recent drug abuse trends in the Seattle-King County area, June 2004; pp. 249–277.

[bibr4-0269881120959615] Belton KJ (1997) Attitude survey of adverse drug-reaction reporting by health care professionals across the European Union. The European Pharmacovigilance Research Group. Euro J Clin Pharmacol 52:423e7. 10.1007/s002280050314 9342576

[bibr5-0269881120959615] Bergman J Wallman P (1998) Promethazine overdose: Is it “Goodnight” after all? N Z Med J 111: 246–248. 9695759

[bibr6-0269881120959615] Bluelight.org (2020) Promethazine. Available at: https://www.bluelight.org/xf/threads/promethazine.598279/ (accessed 14 September 2020).

[bibr7-0269881120959615] Borg J-J Aislaitner G Pirozynski M , et al. (2011) Strengthening and rationalizing pharmacovigilance in the EU: Where is Europe heading to? Drug Safety 34: 187e97. 21332243 10.2165/11586620-000000000-00000

[bibr8-0269881120959615] Burns JM Boyer EW (2013) Antitussives and substance abuse. Subst Abuse Rehabil 4: 75–82. 24648790 10.2147/SAR.S36761 PMC3931656

[bibr9-0269881120959615] Cairns R Brown JA Buckley NA (2016) The impact of codeine re-scheduling on misuse: A retrospective review of calls to Australia’s largest poisons centre. Addiction 111: 1848–1853. 27177599 10.1111/add.13450

[bibr10-0269881120959615] Carney T Wells J Parry CDH , et al. (2018) A comparative analysis of pharmacists’ perspectives on codeine use and misuse: A three country survey. Subst Abuse Treat Prev Policy 13: 12. 29587814 10.1186/s13011-018-0149-2 PMC5870064

[bibr11-0269881120959615] Carr BC (2006) Efficacy, abuse, and toxicity of over-the-counter cough and cold medicines in the pediatric population. Curr Opin Pediatr 18: 184–188. 16601501 10.1097/01.mop.0000193274.54742.a1

[bibr12-0269881120959615] Casati A Sedefov R Pfeiffer-Gerschel T (2012) Misuse of medicines in the European Union: A systematic review of the literature. Euro Addict Res 18: 228e45. 10.1159/000337028 22572594

[bibr13-0269881120959615] Cherian R Westbrook M Ramo D , et al. (2018) Representations of codeine misuse on instagram: Content analysis. JMIR Public Health Surveill 4: e22. 29559422 10.2196/publichealth.8144 PMC5883072

[bibr14-0269881120959615] Chiappini S Schifano F (2016) A decade of gabapentinoid misuse: An analysis of the European Medicines Agency’s ‘suspected adverse drug reactions’ database. CNS Drugs 30:647–654. 27312320 10.1007/s40263-016-0359-y

[bibr15-0269881120959615] Chiappini S Schifano F Corkery JM , et al. (2020) Focus on clozapine withdrawal-and misuse-related cases as reported to the European Medicines Agency (EMA) pharmacovigilance database. Brain Sciences 10: 105. 10.3390/brainsci10020105 PMC7071448 32079135

[bibr16-0269881120959615] Clatts M Giangle LM Goldsamt L , et al. (2010) Nonmedical use of promethazine hydrochloride among heroin injectors in Vietnam: Unrecognized risks and unintended consequences. Subst Use Misuse 45: 515–527. 20141462 10.3109/10826080903452520

[bibr17-0269881120959615] Cookson J (2018) Rapid tranquillisation: The science and advice. B J Psich Advances 24: 346–358.

[bibr18-0269881120959615] Coombes H Cooper RJ (2019) Staff perceptions of prescription and over‑the‑counter drug dependence services in England: A qualitative study. Addiction Science & Clinical. Practice 14: 41. 31718716 10.1186/s13722-019-0170-4 PMC6852756

[bibr19-0269881120959615] Cooper RJ (2013) Over-the-counter medicine abuse-a review of the literature. Journal of Substance Use 18: 82e107. 23525509 10.3109/14659891.2011.615002 PMC3603170

[bibr20-0269881120959615] Corkery JM Schifano F Martinotti G (2020) How deaths can help clinicians and policy-makers understand the risks of novel psychoactive substances. Br J Clin Pharmacol 86: 482–498. 31770457 10.1111/bcp.14183 PMC7080619

[bibr21-0269881120959615] Cowen PJ (1979) Toxic psychosis with antihistamines reversed by physostigmine. Postgrad Med J 55: 556–557. 514934 10.1136/pgmj.55.646.556 PMC2428090

[bibr22-0269881120959615] Dahlman D Abrahamsson T Kral AH , et al. (2016) Nonmedical use of antihistaminergic anxiolytics and other prescription drugs among persons with opioid dependence. J Addict 2016: 9298571. 28097037 10.1155/2016/9298571 PMC5206437

[bibr23-0269881120959615] Delcourt N Jouanjus E Lafaurie M , et al. (2017) Recreational antitussive and antihistamine drug poisoning in adolescents and young adults reported to a French poison Delcourt control center (oral communication abstract). Fund Clin Pharmacol 31: 14.

[bibr24-0269881120959615] Deluca P Davey Z Corazza O , et al. (2002) Identifying emerging trends in recreational drug use; outcomes from the Psychonaut Web Mapping Project. Prog Neuropsychopharmacol Biol Psychiatry 39: 221–226. 10.1016/j.pnpbp.2012.07.011 22841965

[bibr25-0269881120959615] Desai C (2016) Online pharmacies: A boon or bane? Indian J Pharmacol 48: 615–616. 28066095 10.4103/0253-7613.194865 PMC5155458

[bibr26-0269881120959615] Dollberg S Hurvitz H Kerem E , et al. (1989) Hallucinations and hyperthermia after promethazine ingestion. Acta Paediatrica 78: 131–132. 10.1111/j.1651-2227.1989.tb10902.x 2919514

[bibr27-0269881120959615] Drug Enforcement Administration (DEA) (2020) Diversion Control Division. Scheduling Actions Controlled Substances Regulated Chemicals. Available at: https://www.deadiversion.usdoj.gov/schedules/orangebook/orangebook.pdf (accessed 13 April 2020).

[bibr28-0269881120959615] Eickhoff C Hammerlein A Griese N , et al. (2012) Nature and frequency of drug-related problems in self-medication (overthecounter drugs) in daily community pharmacy practice in Germany. Pharmacoepidemiol Drug Saf 21: 254e60. 10.1002/pds.2241 21953893

[bibr29-0269881120959615] Electronic Medicines Compendium (EMC) (2019) Phenergan. Available at: https://www.medicines.org.uk/emc/product/5588/smpc (accessed 25 March 2020).

[bibr30-0269881120959615] Elwood WN (2001) Sticky business: Patterns of procurement and misuse of prescription cough syrup in Houston. J Psychoact Drugs 33(2): 121–133. 10.1080/02791072.2001.10400477 11476259

[bibr31-0269881120959615] Erowid.org (2020) Promethazine. Available at: https://erowid.org/pharms/promethazine/promethazine.shtml (accessed 14 September 2020).

[bibr32-0269881120959615] European Medicines Agency (2010) Note for Guidance—EudraVigilance Human—Processing of Safety Messages and Individual Case Safety Reports (ICSRs) (EMA/H/20665/04/Final Rev. 2). Available at: https://www.ema.europa.eu/en/documents/regulatory-procedural-guideline/note-guidance-eudravigilance-human-processing-safety-messages-individual-case-safety-reports-icsrs_en.pdf (accessed 25 March 2020).

[bibr33-0269881120959615] European Medicines Agency (2013) ICH Guideline E2B (R3) on Electronic Transmission of Individual Case Safety Reports (ICSRs) - Data Elements and Message Specification - Implementation Guide. Available at: https://www.ema.europa.eu/en/documents/scientific-guideline/international-conference-harmonisation-technical-requirements-registration-pharmaceuticals-human-use_en-4.pdf (accessed 26 March 2020).

[bibr34-0269881120959615] European Medicines Agency (2017) Guideline on Good Pharmacovigilance Practices, Module VI—Collection, Management and Submission of Reports of Suspected Adverse Reactions to Medicinal Products (Rev 2). Available at: http://www.ema.europa.eu/docs/en_GB/document_library/Regulatory_and_procedural_guideline/2017/08/WC500232767.pdf (accessed 25 March 2020).

[bibr35-0269881120959615] Evoy KE Covvey JR Peckham AM , et al. (2019) Reports of gabapentin and pregabalin abuse, misuse, dependence, or overdose: An analysis of the Food and Drug Administration Adverse Events Reporting System (FAERS). Res Social Adm Pharm 15: 953–958. 31303196 10.1016/j.sapharm.2018.06.018

[bibr36-0269881120959615] Fingleton N Duncan E Watson M , et al. (2019) Specialist clinicians’ management of dependence on non-prescription medicines and barriers to treatment provision: An exploratory mixed methods study using behavioural theory. Pharmacy 7: 25. 10.3390/pharmacy7010025 PMC6473901 30841493

[bibr37-0269881120959615] Foley M Harris R Rich E , et al. (2015) The availability of over-the-counter codeine medicines across the European Union. Public Health 1465–1470. 26215740 10.1016/j.puhe.2015.06.014

[bibr38-0269881120959615] General Pharmaceutical Council (2019) New Safeguards for People Seeking Medicines Online. Available at: https://www.pharmacyregulation.org/news/new-safeguards-peopleseeking-medicines-online (accessed 26 March 2020).

[bibr39-0269881120959615] Gracious B Abe N Sundberg J (2010) The importance of taking a history of over-the-counter medication use: A brief review and case illustration of ‘PRN’ antihistamine dependence in a hospitalized adolescent. J Child Adolesc Psychopharmacol 20: 521–524. 21186972 10.1089/cap.2010.0031 PMC3025184

[bibr40-0269881120959615] Gummin DD Mowry JB Spyker DA , et al. (2019) 2018 Annual Report of the American Association of Poison Control Centers’ National Poison Data System (NPDS): 36th Annual Report. Clin Toxicol 57: 1220–1413. 10.1080/15563650.2019.1677022 31752545

[bibr41-0269881120959615] Höjer J Tellerup M (2018) Promethazine - an old pharmaceutical that has got a renaissance. An avalanche-like increase in the number of overdose cases in Sweden. Lakartidningen 24: 115. 30040111

[bibr42-0269881120959615] Hou H Yin S Jai S , et al. (2011) Decreased striatal dopamine transporters in codeine-containing cough syrup abusers. Drug Alcohol Depend 118: 148–151. 21477952 10.1016/j.drugalcdep.2011.03.011

[bibr43-0269881120959615] Hughes A Williams MR Lipari RN , et al. (2016) Prescription Drug Use and Misuse in the United States: Results from the 2015 National Survey on Drug Use and Health. Rockville, MD: Substance Abuse and Mental Health Services Administration. Available at: http://www.samhsa.gov/data/ (accessed 25 March 2020). 29792622

[bibr44-0269881120959615] Hughes GF McElnay JC Hughes CM , et al. (1999) Abuse/misuse of non-prescription drugs. Pharm World Sci 21: 251–255. 10658232 10.1023/a:1008788726842

[bibr45-0269881120959615] Ichikura K Okumura Y Takeuchi T (2016) Associations of adverse clinical course and ingested substances among patients with deliberate drug poisoning: A cohort study from an intensive care unit in Japan. PLoS One 11: e0161996. 27560966 10.1371/journal.pone.0161996 PMC4999209

[bibr46-0269881120959615] Jakubowski P Puchała Ł Waldemar G (2018) Recreational use of popular OTC drugs–pharmacological review. Farmacia 66: 209–215.

[bibr47-0269881120959615] Jensen LL Rømsing J Dalhoff K (2017) A Danish survey of antihistamine use and poisoning patterns. Basic Clin Pharmacol Toxicol 120: 64–70. 27288889 10.1111/bcpt.12632

[bibr48-0269881120959615] Jouanjus E Falcou A Deheul S , et al. (2018) Detecting the diverted use of psychoactive drugs by adolescents and young adults: A pilot study. Pharmacoepidemiol Drug Saf 27: 1286–1292. 30255533 10.1002/pds.4624

[bibr49-0269881120959615] Jouanjus E Micallef J Mallaret M , et al. (2019) Comment on: An insight into Z-drug abuse and dependence: An examination of reports to the European Medicines Agency database of suspected adverse drug reactions. Int J Neuropsychopharmacol 22: 528–530. 31194866 10.1093/ijnp/pyz033 PMC6672683

[bibr50-0269881120959615] Khalili H Mohebbi N Hendoiee N , et al. (2012) Improvement of knowledge, attitude and perception of healthcare workers about ADR, a pre- and post-clinical pharmacists’ interventional study. BMJ Open 2: e000367. 10.1136/bmjopen-2011-000367 PMC3278484 22246555

[bibr51-0269881120959615] Klein-Schwartz W (2017) Promethazine Abuse: A Growing Problem? Available at: https://www.mdpoison.com/media/SOP/mdpoisoncom/ToxTidbits/2017/March%202017%20ToxTidbits.pdf (accessed 3 July 2020).

[bibr52-0269881120959615] Leak D Carroll D (1967) Promethazine poisoning: Clinical and electroencephalographic observations. Br Med J 2: 31–32. 6020998 10.1136/bmj.2.5543.31 PMC1841111

[bibr53-0269881120959615] Levine DA (2007) ‘Pharming‘: The abuse of prescription and over-the-counter drugs in teens. Curr Opin Pediatr 19: 270–274. 17505185 10.1097/MOP.0b013e32814b09cf

[bibr54-0269881120959615] Lynch KL Shapiro BJ Coffa D , et al. (2015) Promethazine use among chronic pain patients. Drug Alcohol Depend 150: 92–97. 25754939 10.1016/j.drugalcdep.2015.02.023 PMC4389782

[bibr55-0269881120959615] McCarthy M (2007) Prescription drug abuse up sharply in the USA. Lancet 369: 1505–1506. 17486697 10.1016/S0140-6736(07)60690-4

[bibr56-0269881120959615] McDonough M (2016) Commentary on Cairns et al. Over-the-counter codeine in Australia—questioning the efficacy of current restrictions or re-scheduling. Addiction 111: 1854–1855. 27605083 10.1111/add.13525

[bibr57-0269881120959615] McGee JP Weiss WA (1956) Promethazine, an adjunct to preoperative medication. Ann Surg 144: 861–864. 13373271 10.1097/00000658-195611000-00010 PMC1465261

[bibr58-0269881120959615] McLellan AT Turner BJ (2010) Chronic noncancer pain management and opioid overdose: Time to change prescribing practices. Ann Intern Med 19, 152: 123–124. 20083830 10.7326/0003-4819-152-2-201001190-00012

[bibr59-0269881120959615] Manchikanti L Cash KA Damron KS , et al. (2006) Controlled substance abuse and illicit drug use in chronic pain patients: An evaluation of multiple variables. Pain Physician 9: 215–225. 16886030

[bibr60-0269881120959615] Manchikanti L Sanapati J Benyamin RM , et al. (2018) Reframing the prevention strategies of the opioid crisis: Focusing on prescription opioids, fentanyl, and heroin epidemic. Pain Physician 21: 309–326. 30045589

[bibr61-0269881120959615] Marsden J White M Annand F , et al. (2019) Medicines associated with dependence or withdrawal: A mixed-methods public health review and national database study in England. Lancet Psychiatry 6: 935–950. 31588045 10.1016/S2215-0366(19)30331-1 PMC7029276

[bibr62-0269881120959615] MedDRA (2018) Version 21. Available at: https://www.meddra.org/sites/default/files/guidance/file/smq_intguide_21_0_english.pdf (accessed 25 March 2020).

[bibr63-0269881120959615] Montastruc J-L Sommet A Bagheri H , et al. (2011) Benefits and strengths of the disproportionality analysis for identification of adverse drug reactions in a pharmacovigilance database. Br J Clin Pharmacol 72: 905–908. 21658092 10.1111/j.1365-2125.2011.04037.x PMC3244636

[bibr64-0269881120959615] Monteith S Glenn T (2018) Searching online to buy commonly prescribed psychiatric drugs. Psychiatry Res 260: 248–254. 29220682 10.1016/j.psychres.2017.11.037

[bibr65-0269881120959615] National Institute on Drug Abuse (2014) Cough and Cold Medicine Abuse. Available at: https://www.drugabuse.gov/drugfacts/coughandcoldmedicineabuse (accessed 25 March 2020).

[bibr66-0269881120959615] Novak SP Håkansson A Martinez-Raga J , et al. (2016) Nonmedical use of prescription drugs in the European Union. BMC Psychiatry 16: 274. 27488186 10.1186/s12888-016-0909-3 PMC4972971

[bibr67-0269881120959615] Nutt DJ Blier P (2016) Neuroscience-based Nomenclature (NbN) for Journal of Psychopharmacology. J Psychopharmacol 30: 413–415. 27098017 10.1177/0269881116642903

[bibr68-0269881120959615] O Reilly D Thomas M Moylett E (2015) Cough, codeine and confusion. BMJ Case Rep bcr2015212727. 10.1136/bcr-2015-212727 PMC4691881 26701876

[bibr69-0269881120959615] Orsolini L Francesconi G Papanti D , et al. (2015) Profiling online recreational/prescription drugs’ customers and overview of drug vending virtual marketplaces. Hum Psychopharmacol Clin Exp 30: 302–318. 10.1002/hup.2466 26216567

[bibr70-0269881120959615] Orsolini L Papanti D Corkery J , et al. (2017) An insight into the deep web; why it matters for addiction psychiatry? Hum Psychopharmacol Clin Exp 32: e2573. 10.1002/hup.2573 28657187

[bibr71-0269881120959615] Page CB Duffull SB Whyte IM , et al. (2009) Promethazine overdose: Clinical effects, predicting delirium and the effect of charcoal. QJM: Int J Med 102: 123–131. 10.1093/qjmed/hcn153 19042969

[bibr72-0269881120959615] Parker SD De Gioannis A Page C (2013) Chronic promethazine misuse and the possibility of dependence: A brief review of antihistamine abuse and dependence. J Subst Use 18: 238–241.

[bibr73-0269881120959615] Peacock A Bruno R Gisev N , et al. (2019) New psychoactive substances: Challenges for drug surveillance, control, and public health responses. Lancet 394: 1668–1684. 31668410 10.1016/S0140-6736(19)32231-7

[bibr74-0269881120959615] Peters R Kelder SH Markham CM , et al. (2003) Beliefs and social norms about codeine and promethazine hydrochloride cough syrup (CPHCS) onset and perceived addiction among urban Houstonian adolescents: An addiction trend in the city of lean. J Drug Educ 33: 415–425. 15237866 10.2190/NXJ6-U60J-XTY0-09MP

[bibr75-0269881120959615] Peters R Yacoubian GS Rhodes W (2007) Beliefs and social norms about codeine and promethazine hydrochloride cough syrup (CPHCS) use and addiction among multi-ethnic college students. J Psychoactive Drugs 39: 277–282. 18159781 10.1080/02791072.2007.10400614

[bibr76-0269881120959615] Postigo R Brosch S Slattery J , et al. (2018) EudraVigilance medicines safety database: Publicly accessible data for research and public health protection. Drug Saf 41: 665–675. 29520645 10.1007/s40264-018-0647-1 PMC5990579

[bibr77-0269881120959615] Reeves RR Ladner ME Perry CL , et al. (2015) Abuse of medications that theoretically are without abuse potential. South Med J 108: 151–157. 25772048 10.14423/SMJ.0000000000000256

[bibr78-0269881120959615] Richter PA Burk MP (1992) The potentiation of narcotic analgesics with phenothiazines. J Foot Surg 31: 378–380. 1357024

[bibr79-0269881120959615] Rimsza ME Newberry S (2008) Unexpected infant deaths associated with use of cough and cold medications. Pediatrics 122: e318–322. 18676517 10.1542/peds.2007-3813

[bibr80-0269881120959615] Rose ME (2018) Are prescription opioids driving the opioid crisis? Assumptions vs facts. Pain Med 19: 793–807. 28402482 10.1093/pm/pnx048 PMC6018937

[bibr81-0269881120959615] Saatcioglu O Evren C (2005) A case of pheniramine dependence. Subst Abuse 26: 45–47. 10.1300/j465v26n01_06 16492663

[bibr82-0269881120959615] Schifano F (2020) Coming off prescribed psychotropic medications: Insights from their use as recreational drugs. Psychother Psychosom 89: 274–282. 32615566 10.1159/000507897

[bibr83-0269881120959615] Schifano F Chiappini S (2018a) Is there such a thing as a ‘lope’ dope? Analysis of loperamide-related European Medicines Agency (EMA) pharmacovigilance database reports. PLoS One 13: e0204443. 30286103 10.1371/journal.pone.0204443 PMC6171858

[bibr84-0269881120959615] Schifano F Chiappini S (2018b) Is there a potential of misuse for venlafaxine and bupropion? Front Pharmacol 9: 231. 29618978 10.3389/fphar.2018.00239 PMC5871746

[bibr85-0269881120959615] Schifano F Chiappini S (2019) Pregabalin: A range of misuse-related unanswered questions. CNS Neurosci Ther 25: 659–660. 30834646 10.1111/cns.13115 PMC6488882

[bibr86-0269881120959615] Schifano F Chiappini S Corkery JM , et al. (2019a) An insight into Z-drug abuse and dependence: An examination of reports to the European Medicines Agency Database of Suspected Adverse Drug Reactions. Int J Neuropsychopharmacol 22: 270–277. 30722037 10.1093/ijnp/pyz007 PMC6441128

[bibr87-0269881120959615] Schifano F Chiappini S Corkery JM , et al. (2019b) Assessing the 2004–2018 fentanyl misusing issues reported to an international range of adverse reporting systems. Front Pharmacol 10: 46. 30774595 10.3389/fphar.2019.00046 PMC6367955

[bibr88-0269881120959615] Schifano F Leoni M Martinotti G , et al. (2003). Importance of cyberspace for the assessment of the drug abuse market: Preliminary results from the Psychonaut 2002 project. Cyberpsychol Behav 6: 405–410. 14511453 10.1089/109493103322278790

[bibr89-0269881120959615] Schwan S Sundström A Stjernberg E , et al. (2010) A signal for an abuse liability for pregabalin–results from the Swedish spontaneous adverse drug reaction reporting system. Euro J Clin Pharmacol 66: 947–953. 10.1007/s00228-010-0853-y 20563568

[bibr90-0269881120959615] Scott J Pache D Keane G , et al. (2007) Prolonged anticholinergic delirium following antihistamine overdose. Australas Psychiatry 15: 242–244. 17516189 10.1080/10398560601147020

[bibr91-0269881120959615] Shapiro BJ Lynch KL Toochinda T , et al. (2013) Promethazine misuse among methadone maintenance patients and community-based injection drug users. J Addict Med 7: 96–101. 23385449 10.1097/ADM.0b013e31827f9b43 PMC3618500

[bibr92-0269881120959615] Sharma A Hamelin BA (2003) Classic histamine H1 receptor antagonists: A critical review of their metabolic and pharmacokinetic fate from a bird’s eye view. Curr Drug Metab 4: 105–129. 12678691 10.2174/1389200033489523

[bibr93-0269881120959615] Shields LB Hunsaker JC Corey TS , et al. (2007) Methadone toxicity fatalities: A review of medical examiner cases in a large metropolitan area. J Forensic Sci 52: 1389–1395. 18093068 10.1111/j.1556-4029.2007.00565.x

[bibr94-0269881120959615] Soussan C Andersson M Kjellgren A (2018) The diverse reasons for using Novel Psychoactive Substances: A qualitative study of the users’ own perspectives. Int J Drug Policy 52: 71–78. 29241144 10.1016/j.drugpo.2017.11.003

[bibr95-0269881120959615] Speeg KV Wang S Avant GR , et al. (1981) In vitro antagonism of benzodiazepine binding to cerebral receptors by H1 and H2 antihistamines. J Lab Clin Med 97:112–122. 6108979

[bibr96-0269881120959615] Substance Abuse and Mental Health Services Administration (2008). The NSDUH Report – Misuse of Over-the-Counter Cough and Cold Medications among Persons Aged 12 to 25. Rockville, MD: SAMHSA, Office of Applied Studies.

[bibr97-0269881120959615] Tang AK Tang WK Liang HJ , et al. (2012) Clinical characteristics of cough mixture abusers referred to three substance abuse clinics in Hong Kong: A retrospective study. East Asian Arch Psychiatry 22: 154–159. 23271584

[bibr98-0269881120959615] Thomas A Nallur DG Jones N , et al. (2008) Diphenhydramine abuse and detoxification: A brief review and case report. J Psychopharmacol 23: 101–105. 18308811 10.1177/0269881107083809

[bibr99-0269881120959615] Throckmorton DC Gottlieb S Woodcock J (2018) The FDA and the next wave of drug abuse - proactive pharmacovigilance. N Engl J Med 379: 205–207. 29847203 10.1056/NEJMp1806486

[bibr100-0269881120959615] Timnak C Gleason O Okla T (2004) Promethazine-induced psychosis in a 16-year-old girl. Psychosomatics 45: 89–90. 14709767 10.1176/appi.psy.45.1.89

[bibr101-0269881120959615] TMZ (2014) Bad news for Justin, Soulja famous ‘sizzurp’ cough syrup yanked from market. Available at: https://www.tmz.com/2014/04/23/sizzurp-cough-syrup-off-market-justin-bieber-lean-codeine-actavis/ (accessed 14 July 2020).

[bibr102-0269881120959615] Tobin CL Dobbin M McAvoy B (2013) Regulatory responses to over-the-counter codeine analgesic misuse in Australia, New Zealand and the United Kingdom. Aust N Z J Public Health 37: 483–488. 24090333 10.1111/1753-6405.12099

[bibr103-0269881120959615] Tsay ME Procopio G Anderson BD , et al. (2015) Abuse and intentional misuse of promethazine reported to US Poison Centers 2002 to 2012. J Addict Med 9: 233–237. 25822213 10.1097/ADM.0000000000000124

[bibr104-0269881120959615] United Nations Office on Drugs and Crime (UNODC) (2013) The non-medical use of prescription drugs. Policy direction issues. Discussion Paper. Available at: https://www.unodc.org/documents/drug-prevention-and-treatment/nonmedical-use-prescription-drugs.pdf (accessed 3 July 2020).

[bibr105-0269881120959615] Van Hout M Norman I (2016) Misuse of non-prescription codeine containing products: Recommendations for detection and reduction of risk in community pharmacies. Int J Drug Policy 27: 17–22. 26454626 10.1016/j.drugpo.2015.09.007

[bibr106-0269881120959615] Wazaify M Shields E Hughes CM , et al. (2005) Societal perspectives on over-the-counter (OTC) medicines. Fam Prac 22: 170–176. 10.1093/fampra/cmh723 15710640

[bibr107-0269881120959615] Wells JSG Bergin M Van Hout MC , et al. (2018) Purchasing Over The Counter (OTC) medicinal products containing codeine: Easy access, advertising, misuse and perceptions of medicinal risk. J Pharm Pharm Sci 21: 286–295. 10.18433/jpps30049 30011259

